# Editorial: Overcoming therapeutic resistance in head and neck squamous cell carcinoma

**DOI:** 10.3389/fonc.2025.1611819

**Published:** 2025-06-05

**Authors:** Souvick Roy, Prakriti Sen, Madhabananda Kar, Birendranath Banerjee

**Affiliations:** ^1^ Moores Cancer Center, University of California, San Diego, La Jolla, CA, United States; ^2^ Department of Surgical Oncology, All India Institute of Medical Sciences, Bhubaneswar, Odisha, India; ^3^ All India Institute of Medical Sciences, Darbhanga, Bihar, India; ^4^ inDNA Centre for Research and Innovation in Molecular Diagnostics, inDNA Life Sciences Private Limited, Bhubaneswar, Odisha, India; ^5^ JBS Haldane Centre of Molecular Medicine, Silicon University, Bhubaneswar, Odisha, India

**Keywords:** head and neck squamous cell carcinoma, therapy resistance, molecular biomarker, cancer stem cells, novel therapeutics

Head and neck squamous cell carcinoma (HNSCC) is one of the most prevalent cancers worldwide and comprises at least two forms with distinct etiologies: human papillomavirus (HPV) positive and HPV -negative (1). The majority of HNSCCs primarily originate from the squamous cells lining the tissues of the oral cavity, pharynx, larynx, nasal cavity, paranasal sinuses and salivary glands (1, 2). The HNSCCs of the oral cavity are primarily associated with tobacco (smoked or chewed), excessive alcohol consumption and areca nut (betel quid). However, HNSCCs originating in the oropharynx and hypopharynx have been attributed to HPV infection, primarily HPV-16 (1, 2). The standard-of-care for treating head and neck squamous cell carcinoma (HNSCC) involves surgical resection of the primary tumor, followed by adjuvant radiotherapy, platinum-based chemotherapy (cisplatin or carboplatin), targeted therapy with cetuximab, or a combination of these treatment modalities (1–4). In 2016, FDA approved immune check-point inhibitor nivolumab and pembrolizumab for treatment of cisplatin-refractory recurrent or metastatic HNSCC patients whereas for patients who present with unresectable or metastatic disease, pembrolizumab is approved as first-line therapy (5–7). Despite recent advancements, acquired resistance to these treatment modalities remains a major challenge in the treatment of HNSCC (8–10). Patients with recurrent or metastatic disease have limited treatment options and poor 5-year survival rates (9). Therefore, there is an unmet and urgent need to identify molecular biomarkers associated with therapeutic resistance and to elucidate their potential molecular mechanisms. The Research Topic *“Overcoming Therapeutic Resistance in Head and Neck Squamous Cell Carcinoma”* aims to identify molecular biomarkers and their underlying molecular mechanisms in promoting therapeutic resistance in HNSCC. In addition, this Research Topic also focuses on the development of novel therapeutic strategies and their clinical application in HNSCC patients to overcome therapeutic resistance.

It is well known that HPV positive HNSCC patients typically have better outcomes compared to HPV negative HNSCC patients, partly due to their intrinsic sensitivity to radiation (10–12). The small-molecule kinase inhibitors (smKIs) targeting DNA double-strand breaks and single-strand breaks repair proteins, namely, Ataxia telangiectasia mutated (ATM) or Ataxia telangiectasia mutated and Rad3-related (ATR) kinases, have emerged as an attractive treatment option for the treatment of HNSCC (13–15). In this Research Topic, Meidenbauer et al., explored the impact of combining ATM or ATR inhibitors with hypofractionated radiotherapy (RT) in influencing the immunogenicity of Head and neck squamous cell carcinoma cells. The authors observed that combined treatment of ATM or ATR inhibitor with hypofractionated radiotherapy promoted increased cell death compared to individual treatment with ATM or ATR inhibitors irrespective of HPV status. Furthermore, RT+ ATRi treatment led to upregulation of the immune-stimulatory phenotype to promote anti-tumor immune response. The authors showed a differential expression pattern of pro-inflammatory cytokines between RT+ ATRi and RT+ ATMi treatment in HNSCC cells where RT+ATRi promoted increased expression of pro-inflammatory cytokines such as IL-6, IL-8, TNF-α etc. In contrast, RT+ATMi promoted an immunosuppressive phenotype and led to downregulation of the RIG-I signaling pathway, a cytoplasmic sensing pathway of nucleic acids (16) and upregulation of EDIL3, a known anti-inflammatory factor and immune modulator triggering ‘immune execution’ (17, 18). Although the strategy may be attractive with a scope of lower radiation dosage but further investigation is required to determine the efficacy of combined treatment of ATM or ATR inhibitors with RT in clinical settings.

In this Research Topic, Li et al. presented a case of a 78-year-old man diagnosed with human papillomavirus (HPV+) and programmed death ligand 1 (PD-L1+) tonsillar squamous cell carcinoma (TSCC). TSCC represents 15%-20% of oropharyngeal squamous cell carcinoma (OPSCC) cases and the majority of them are HPV+ (19). The multimodal treatment strategies such as surgery, chemotherapy, radiation and immunotherapy showed favorable outcomes in TSCC patients as compared to single modality treatment (20). In this case report, the patient was treated with intravenous administration of Camrelizumab, a PD-1 inhibitor and gemcitabine (GEM), a chemotherapeutic drug. After 8 cycles of treatment the patient exhibited a partial response (PR) with a moderate increase in tumor size whereas lymph node size was decreased. Subsequently, concurrent treatment with radiotherapy and intravenous nab-paclitaxel led to a complete response with no evidence of recurrence or metastasis after a 12-month follow-up. This study provides evidence that Camrelizumab plus GEM followed by concurrent chemoradiotherapy may be a promising therapeutic strategy for HPV-positive and PD-L1-positive TSCC patients.

In another interesting case report, Otsuka et al. presented the case of a 65-year-old man with a history of smoking who was diagnosed with HNSCC of occult primary origin classified as T0N3bM0, stage IVB with programmed cell death-ligand 1 expression. Immune -related adverse events (irAEs) including cytokine release syndrome (CRS) rarely develop after nivolumab treatment but can be severe and lead to multi-organ failure (21, 22). In this study, the patient experienced repeated recurrence of Immune checkpoint inhibitor (ICI) induced CRS. The patient presented with high fever and hypotension, along with an elevated C-reactive protein level, suggesting the possibility of septic shock. However, all bacterial cultures performed from sputum, urine, and blood were negative. The diagnosis of nivolumab-induced CRS was confirmed based on elevated serum levels of ferritin and IL-6. The patient responded well to high-dose steroids such as methylprednisolone. However, CRS recurred during steroid tapering and coincided with an increased tumor burden. Nevertheless, it was successfully managed with an increased dose of methylprednisolone. The patient’s general health condition was poor and eight months post-discontinuation of nivolumab, the patient died due to cancer progression. This case report suggests that early detection and treatment with steroids are essential in the management of CRS.

Locally advanced hypopharyngeal squamous cell carcinoma (LA HPSCC) is one of the most malignant tumors of the head and neck, with a high recurrence rate and poor prognosis (23–26). In this retrospective, single-arm, single center clinical trial led by Gao et al. the authors compared the clinical efficacy and safety between PD-1 inhibitors, pembrolizumab, plus TP regimen (paclitaxel liposome + nedaplatin) and targeted therapy of nimotuzumab, a monoclonal antibody against EGFR plus TP regimen among LA HPSCC patients in the neoadjuvant setting. Post neoadjuvant treatment, the response was assessed based on RECIST criteria. It was observed that in both the treatment groups, the objective response rate (ORR) and disease control rate (DCR) were similar with no significant differences, but the pathological complete response (pCR) rate was much higher in Group A than in Group B. Furthermore, the rate of primary tumor downstaging and 1-year overall survival were much higher in Group A LA HPSCC patients. In addition, the larynx function preservation rate was significantly higher in Group A patients and both treatment strategies did not exhibit a significant difference in the incidence of grade 3-4 treatment-related adverse events (TRAEs). These results suggest that PD-1 inhibitors combined with chemotherapy in the neoadjuvant setting showed better short-term efficacy compared to targeted therapy and exhibit a trend toward improved long-term survival in LA HPSCC patients.

In another interesting pilot clinical trial conducted by Yoon et al. the authors explored the efficacy and safety of the topical toll-like receptor-7 (TLR-7) agonist, imiquimod, in the neoadjuvant setting in patients with early-stage oral squamous cell carcinoma (OSCC). Previous reports suggest that Imiquimod exerts an anti-tumor activity by stimulating innate and adaptive immunity and promoting tumor cell apoptosis (25, 27, 28). In this single-arm, open label pilot study, imiquimod 5% cream was self-administered to early-stage OSCC patients (clinical T1 and T2 without nodal involvement) for 28 days followed by surgical resection of the tumor. It was observed that 60% of early-stage OSCC patients experienced a >50% reduction in tumor cell count following imiquimod treatment. It was also observed that 60% of patients had an immune-related major pathologic response (irMPR) with two patients reporting a complete pathologic response, and 40% of patients had a partial response (PR) with the percent residual viable tumor ranging from 25% to 65%. It was observed that helper and cytotoxic T-cells significantly contributed to the reduction in the tumor. The treatment was well tolerated at the application site, and no patients experienced grade 4 life-threatening events. The median follow-up time was 17 months for 14 patients, and 13 (93%) out of 14 patients had a 1-year recurrence-free survival after neoadjuvant imiquimod therapy. This study highlights that neoadjuvant imiquimod immunotherapy can be a safe and promising treatment option for early-stage OSCC.

This Research Topic also includes a review article summarizing the clinical application of exosomal circular RNA in squamous cell carcinoma. CircRNAs are single-stranded non-coding-RNAs that regulate gene expression, and are easily detectable in blood, rendering them ideal candidates as non-invasive biomarkers (29, 30). Tumor-derived exosomal cargo containing CircRNAs offers an attractive platform for the development of cancer-specific diagnostic and prognostic biomarkers and is likely to provide mechanistic insights for the identification of therapeutic targets (31, 32). In this review article by Wang et al. the authors summarized the biological functions of exosomal circRNAs, their expression pattern and their role in promoting metastasis and therapy resistance in squamous cell carcinoma (SCC). In addition, the authors also reviewed the existing literature highlighting the exosomal circRNAs as diagnostic and prognostic biomarkers along with their potential as a therapeutic target in SCC.

In summary this Research Topic represents an important and diverse contribution in the field of head and neck cancer with novel and unique treatment strategies to overcome therapy resistance in HNSCC. Finally, this Research Topic includes a review article summarizing the clinical significance of exosomal circular RNA in the pathogenesis of SCC and its utility for clinical applications. The field of HNSCC research and the quest to find novel therapeutic strategies for effectively overcoming resistance and minimizing the chance of recurrence to increase disease-free survival are growing rapidly. We are hopeful that the advent of new biomarkers along with the clinical efficacy of new molecules will lead to a decrease in the disease burden in the coming decade.
